# Supplementary material for: Influence of Hydrogenation on Morphology, Chemical Structure and Photocatalytic Efficiency of Graphitic Carbon Nitride
Source: Int J Mol Sci. 2021 Dec 3;22(23):13096. doi: 10.3390/ijms222313096 (PMC8657794; doi:10.3390/ijms222313096)
Supplement: Supplementary file 1 [file ijms-22-13096-s001.zip › ijms-1477104-supplementary.pdf]

International Journal of
Molecular Sciences

MDPI

Supplementary Materials

Influence of hydrogenation on morphology, chemical structure and photocatalytic efficiency of graphitic carbon nitride

Daria Baranowska *, Tomasz Kędzierski, Małgorzata Aleksandrzak, Ewa Mijowska and Beata Zielińska *

Department of Nanomaterials Physicochemistry, Faculty of Chemical Technology and Engineering, West Pomeranian University of Technology, Szczecin, Piastow Ave. 42, 71-065 Szczecin, Poland; tomasz.kedzierski@zut.edu.pl (T.K.); malgorzata.wojtoniszak@zut.edu.pl (M.A.); ewa.borowiak-palen@zut.edu.pl (E.M.)

* Correspondence: bd36293@zut.edu.pl (D.B.); bzielinska@zut.edu.pl (B.Z.)

Citation: Baranowska, D.; Kędzierski, T.; Aleksandrzak, M.; Mijowska, E.; Zielińska, B. Influence of Hydrogenation on Morphology, Chemical Structure and Photocatalytic Efficiency of Graphitic Carbon Nitride. *Int. J. Mol. Sci.* **2021**, *22*, 13096. https://doi.org/10.3390/ijms222313096

Academic Editor: Valentina Villari

Received: 8 November 2021
Accepted: 1 December 2021
Published: 3 December 2021

Publisher's Note: MDPI stays neutral with regard to jurisdictional claims in published maps and institutional affiliations.

Copyright: © 2021 by the authors. Licensee MDPI, Basel, Switzerland. This article is an open access article distributed under the terms and conditions of the Creative Commons Attribution (CC BY) license (https://creativecommons.org/licenses/by/4.0/).

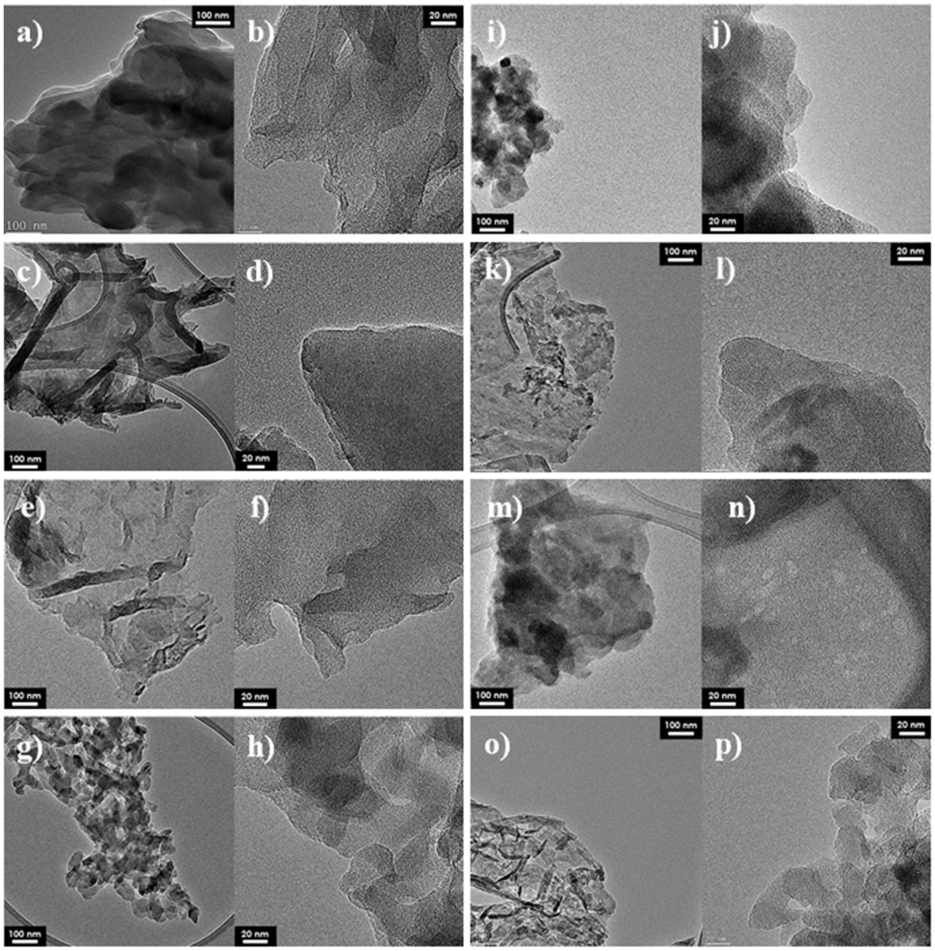

Figure S1. TEM images of gCN (**a**,**b**), 400-2 (**c**,**d**), 450-2 (**e**,**f**), 550-2 (**g**,**h**), 500-1 (**i**,**j**), 500-2 (**k**,**l**), 500-3 (**m**,**n**), and 500-4 (**o**,**p**).

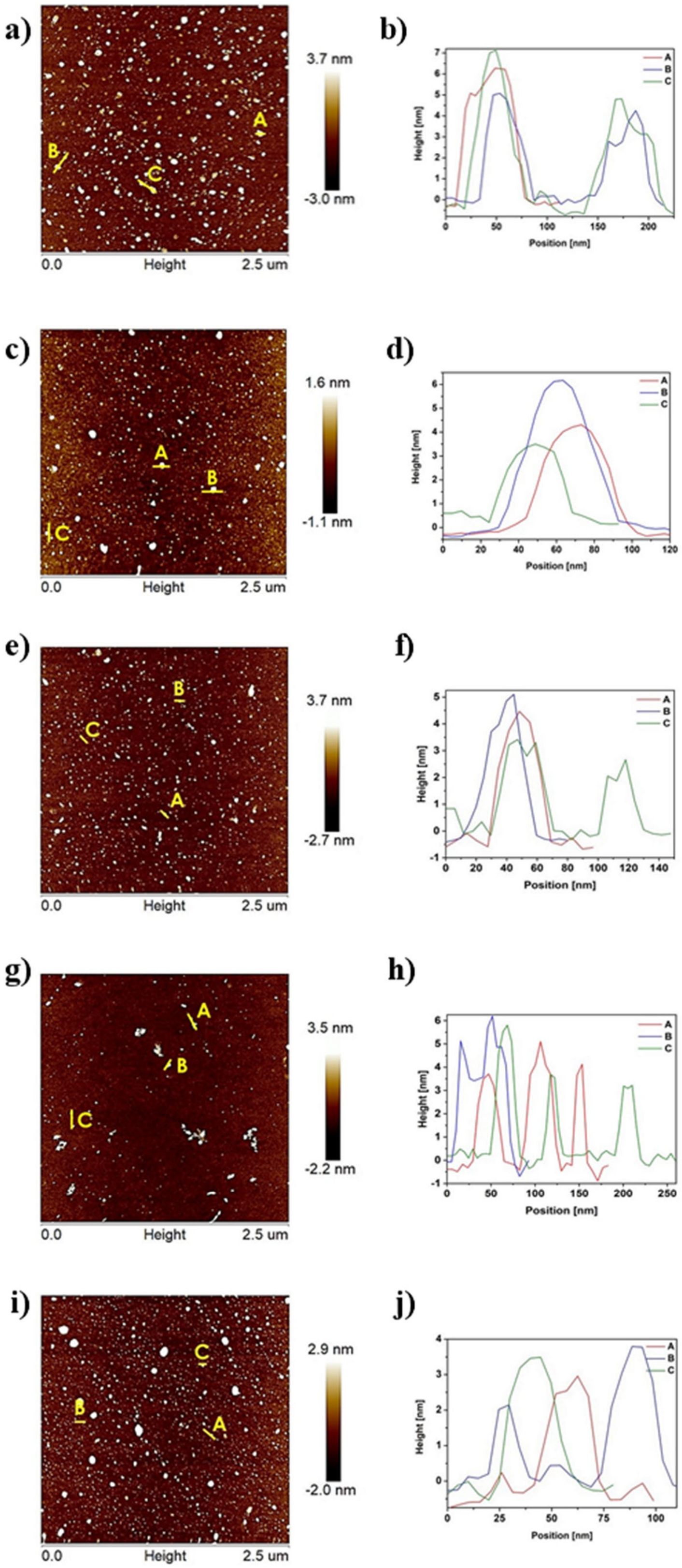

Figure S2. AFM images and height profiles of 400-2 (**a**,**b**), 450-2 (**c**,**d**), 500-1 (**e**,**f**), 500-3 (**g**,**h**), and 550-2 (**i**,**j**).

Table S1. AFM data of gCN prepared under different conditions.

Sample	Thickness [nm]	Mean thickness [nm]	Lateral size [μm]	Mean size [μm]	Number of layers
gCN	4 – 9	6.46	0.067 – 0.136	0.104	12 – 28
400-2	4 – 8	5.82	0.062 – 0.088	0.074	12 – 25
450-2	3 – 6	4.34	0.061 – 0.099	0.073	9 – 18
500-1	2 – 7	4.26	0.044 – 0.074	0.055	6 – 21
500-2	1 – 5	2.59	0.031 – 0.133	0.092	3 – 15
500-3	1 – 5	2.24	0.028 – 0.077	0.049	3 – 15
500-4	1 – 5	2.28	0.036 – 0.098	0.075	3 – 15
550-2	2 – 6	4.55	0.044 – 0.098	0.058	6 – 22

Table S2. Carbon (C), nitrogen (N), oxygen (O) atomic concentration in gCN and 500-4.

Sample	C (at%)	N (at%)	O (at%)
gCN	37.09	62.46	0.45
500-4	35.16	64.73	0.12

Table S3. Chemical composition of gCN and 500-4 calculated from the peak-fitting procedure applied to the C 1s and N 1s spectra of the samples.

Sample	N – C = N (at%)	C – NH_x (at%)	C – C/C = C (at%)	N_2C (at%)	N – H_x (at%)	N_3C (at%)
gCN	79.70	8.40	11.9	81.02	4.04	14.94
500-4	72.75	21.66	5.60	88.41	9.22	2.37

Table S4. Kinetic parameters of photocatalytic RhB decomposition.

Sample	k [1/min]	R^2
gCN	0.27314	0.98761
400-2	0.47160	0.99391
450-2	0.46404	0.99498
500-1	0.46198	0.98105
500-2	0.80280	0.98976
500-3	0.79782	0.99592
500-4	1.11803	0.99253
550-2	0.69168	0.99351

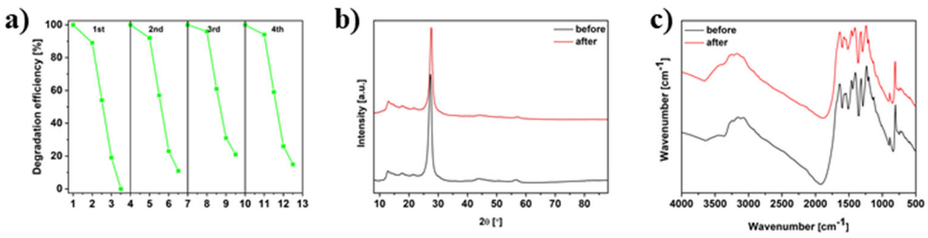

Figure S3. 500-4 photocatalyst stability (**a**). The XRD patterns (**b**) and FTIR spectra (**c**) before (black line) and after (red line) four photocatalytic cycles of RhB decomposition catalyzed by 500-4 sample.

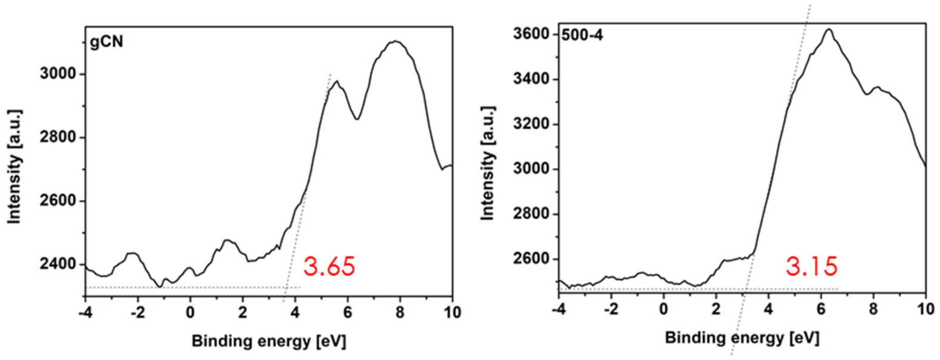

Figure S4. XPS valance band spectra of the gCN and 500-4 sample.
